# Effects of the synergistic treatments of arbuscular mycorrhizal fungi and trehalose on adaptability to salt stress in tomato seedlings

**DOI:** 10.1128/spectrum.03404-23

**Published:** 2024-01-23

**Authors:** Gongshan Chen, Anna Yang, Jianzhong Wang, Lixia Ke, Shaoxing Chen, Wentao Li

**Affiliations:** 1 Anhui Provincial Key Lab of the Conservation and Exploitation of Biological Resources, Anhui Normal University, Wuhu, China; Connecticut Agricultural Experiment Station, Connecticut, USA

**Keywords:** synergistic effects, arbuscular mycorrhizal fungi, trehalose, salt stress, antioxidant system

## Abstract

Arbuscular mycorrhizal fungi (AMF) could establish symbiosis with plant roots, which enhances plant resistance to various stresses, including drought stress and salt stress. Besides AMF, chemical stimulants such as trehalose (Tre) can also play an important role in helping plants alleviate damage of adversity. However, the mechanism of the effect of AMF combined with chemicals on plant stress resistance is unclear. The objective of this study was to explore the synergistic effects of *Claroideoglomus etunicatum* AMF and exogenous Tre on the antioxidant system, osmoregulation, and resistance-protective substance in plants in response to salt stress. Tomato seedlings were inoculated with *Claroideoglomus etunicatum* and combined with exogenous Tre in a greenhouse aseptic soil cultivation experiment. We measured the arbuscular mycorrhizal symbiont development, organic matter content, and antioxidant enzyme activity in tomato seedlings. Both AMF and Tre improved the synthesis of chlorophyll content in tomato seedlings; regulated the osmotic substance including soluble sugars, soluble protein, and proline of plants; and increased the activity of superoxide dismutase, peroxidase, and catalase. The combination of AMF and Tre also reduced the accumulation of malondialdehyde and alleviated the damage of harmful substances to plant cells in tomato seedlings. We studied the effects of AMF combined with extraneous Tre on salt tolerance in tomato seedlings, and the results showed that the synergistic treatment of AMF and Tre was more efficient than the effects of AMF inoculation or Tre spraying separately by regulating host substance synthesis, osmosis, and antioxidant enzymes. Our results indicated that the synergistic effects of AMF and Tre increased the plant adaptability against salt damage by enhancing cell osmotic protection and cell antioxidant capacity.

**IMPORTANCE:**

AMF improve the plant adaptability to salt resistance by increasing mineral absorption and reducing the damage of saline soil. Trehalose plays an important role in plant response to salt damage by regulating osmotic pressure. Together, the use of AMF and trehalose in tomato seedlings proved efficient in regulating host substance synthesis, osmosis, and antioxidant enzymes. These synergistic effects significantly improved seedling adaptability to salt stress by enhancing cell osmotic protection and cell antioxidant capacity, ultimately reducing losses to crops grown on land where salinization has occurred.

## INTRODUCTION

Soil salinization has become one of the major environmental issues globally and one of the main causes of crop loss. It is estimated that salinization is predicted to threaten half of the world’s arable land in the next three decades (1, 2). Salinization causes difficulty in seed germination (3), affects the growth and development, decreases nutrient accumulation in plants, and reduces crop yields (4). In addition to damaging plants, salinization also affects the microbial community structure in the soil (5–7). Tomato, one of the most often cultivated vegetable species worldwide, is vulnerable to various kinds of stress in the actual production process such as salt stress, high-temperature stress, and drought stress. Studies confirmed that salt affects tomato enzyme activity and hormone levels when the seeds sprout (8, 9).

Arbuscular mycorrhizal fungi (AMF) could establish mutualistic symbionts with plant roots, which is a channel of material exchange and information exchange between host plants and soil. They are involved in biogeochemical cycles of carbon and nitrogen and improve the host’s absorption of mineral elements in the soil, such as N, P, and K (10–12). AMF improve plant stress resistance as the ecological environmental protection agent. Mutualistic symbionts significantly improve host chlorophyll content, mineral absorption, and assimilation by inoculating with AMF (3, 13–15). Besides, AMF alleviate fungal diseases in plants, such as the Black foot disease in vines (16). Mycorrhizal symbionts also indirectly reduce the damage of salt to plants because mycorrhiza selectively absorb ions, control the input of Na^+^, and reduce the content of Na^+^ in plants under the salt condition (3, 12, 16–18). Therefore, inoculating the host plants with AMF is an effective way to enhance plant development, promote substance accumulation (19, 20), and improve plant endurance (21–25).

Exogenous plant stimulants also increase crop yield and resistance to different environmental stress factors. Melatonin promotes cotton seed germination and enhances drought and salt resistance of cotton by promoting root development and prolonging root life (26–28). Citric acid inhibits oxidative stress and improves the tolerance of tomato to chromium (29). Trehalose (Tre) is a kind of natural, non-reducing disaccharide that exists in lower plants and animals. Some studies have shown that lack of trehalose in microorganisms hinders the development of spores and mycelia (30). A number of reviews in recent years have indicated that Tre has a non-specific protective effect on biological activity and significantly mitigates the damage caused by reduced moisture (31, 32). Xie et al. observed that application of Tre to tomatoes improved salt stress tolerance by enhancing enzyme activity associated with the Tre metabolic pathway (33). The synergistic effects of endogenous trehalose and NO improved the tolerance of capsicum to cadmium (29). Under osmotic stress, many plants, including tomatoes, respond to stress by accumulating Tre (34–36). Tre accumulation in rice is promoted by modifying the trehalose hexaphosphate synthase gene, which then improves the stress resistance of rice (37). Previous studies have reported on just individual treatments of either AMF or Tre on plants, while in this study, the synergistic effects of AMF and Tre as a combined treatment were also analyzed. We further focused on the joint action of AMF and Tre on the antioxidant system, osmoregulation, and resistance-protective substance under salt stress condition.

## RESULTS

### Mycorrhizal symbiosis, growth, and development in tomato seedlings under salt stress

**Fig 1:**
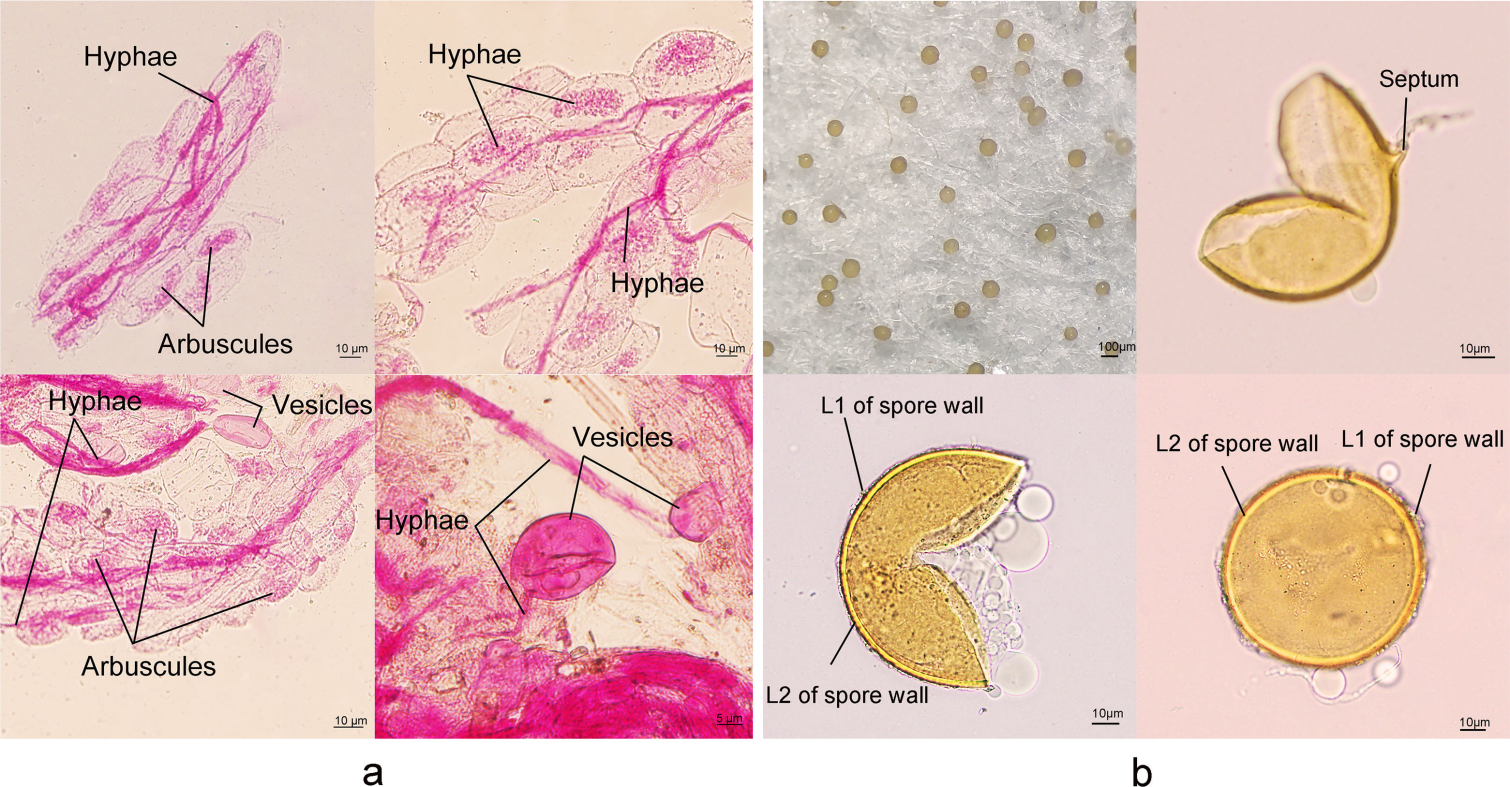
Mycorrhizal structure of a tomato seedling colonized with *C. etunicatum* fungi (a) and *C. etunicatum* fungal spores mounted with polyvinyl lactic acid glycerol (b).

Typical mycorrhizal symbiont structures were detected in all tomato roots inoculated with *Claroideoglomus etunicatum*. The extraradical mycelium penetrated into tomato roots and extended through longitudinal intercellular air spaces. An arbuscule branched from an intercellular hypha and developed numerous fine-branched high hyphae, which was limited in a cortex cell (Fig. 1a).

Spores were separated from the soil and mounted with polyvinyl lactic acid glycerol. Two layers (L1 and L2) of the spore wall and a bridging structure that resembles a septum were observed, which locates the innermost sublayer of the laminate layer of the spore wall in *C. etunicatum* (Fig. 1b).

**Fig 2:**
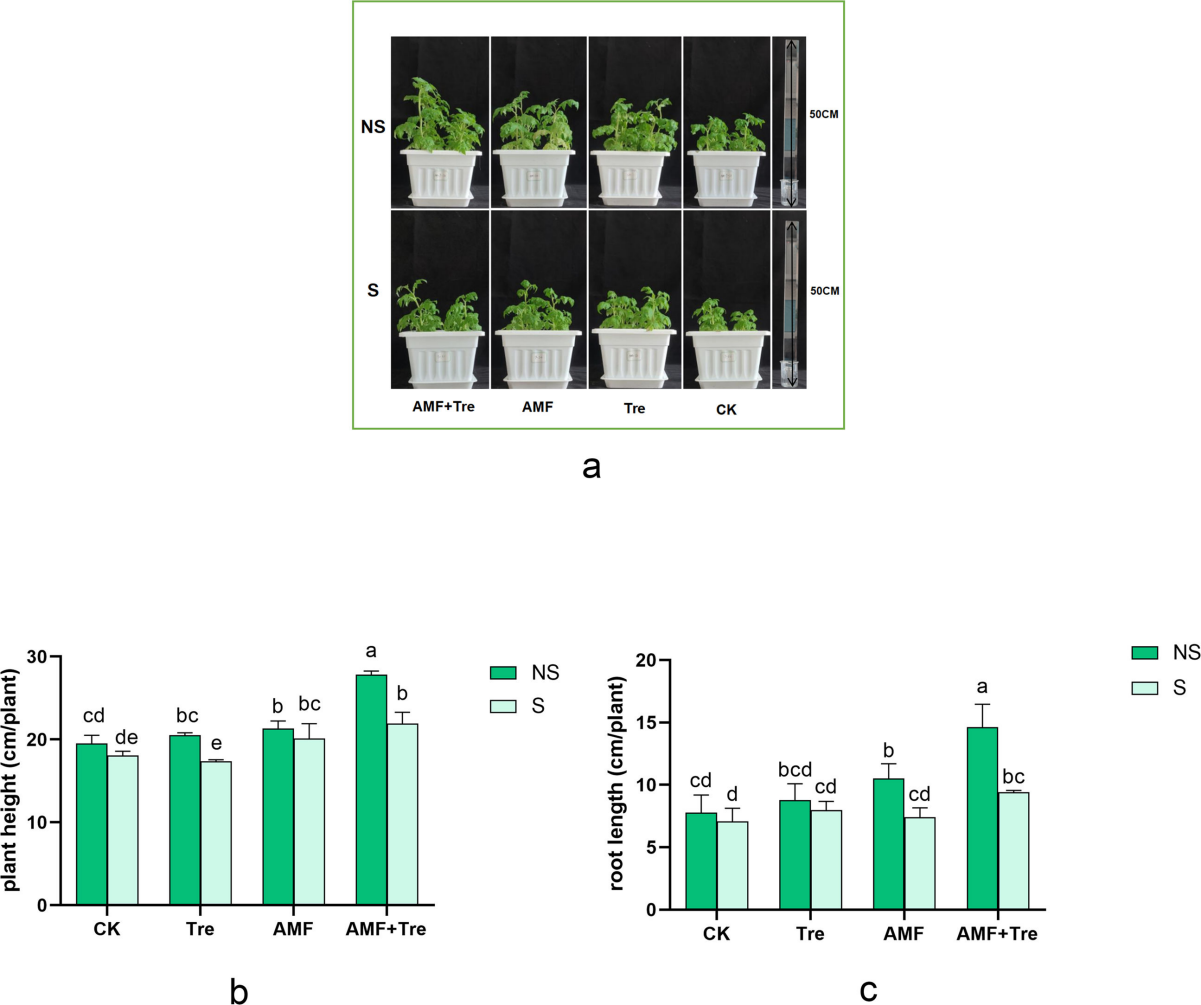
Effects of different treatments on plant development: (a) plant height (b) and root length (c) under non-salt stress (NS) and salt stress (S). CK: tomato seedlings without stimulants; Tre: spraying tomato seedlings with trehalose; AMF: inoculated with *C. etunicatum*; AMF + Tre: synergistic treatment of AMF and Tre. The data were expressed as “mean ± standard error” (*n* = 3), and significant differences were measured by Duncan’s test (*P* < 0.05).

Salt stress inhibited plant development in tomato seedlings, while AMF treatment and Tre treatment relieved this inhibition (Fig. 2a through c). The AMF + Tre treatment increased the beneficial effect and improved the growth of the plant significantly (Fig. 2a). Multi-factor analysis of variance (ANOVA) showed that AMF significantly increased plant height under salt stress (*P* < 0.05), and the efficiency was improved by AMF + Tre treatment. Plant height (21.93 cm) and root length (9.40 cm) in the tomato seedlings treated with AMF + Tre were the highest, showing an increase of 21.36% and 32.96%, separately (*P* < 0.05) (Fig. 2c).

**Fig 3:**
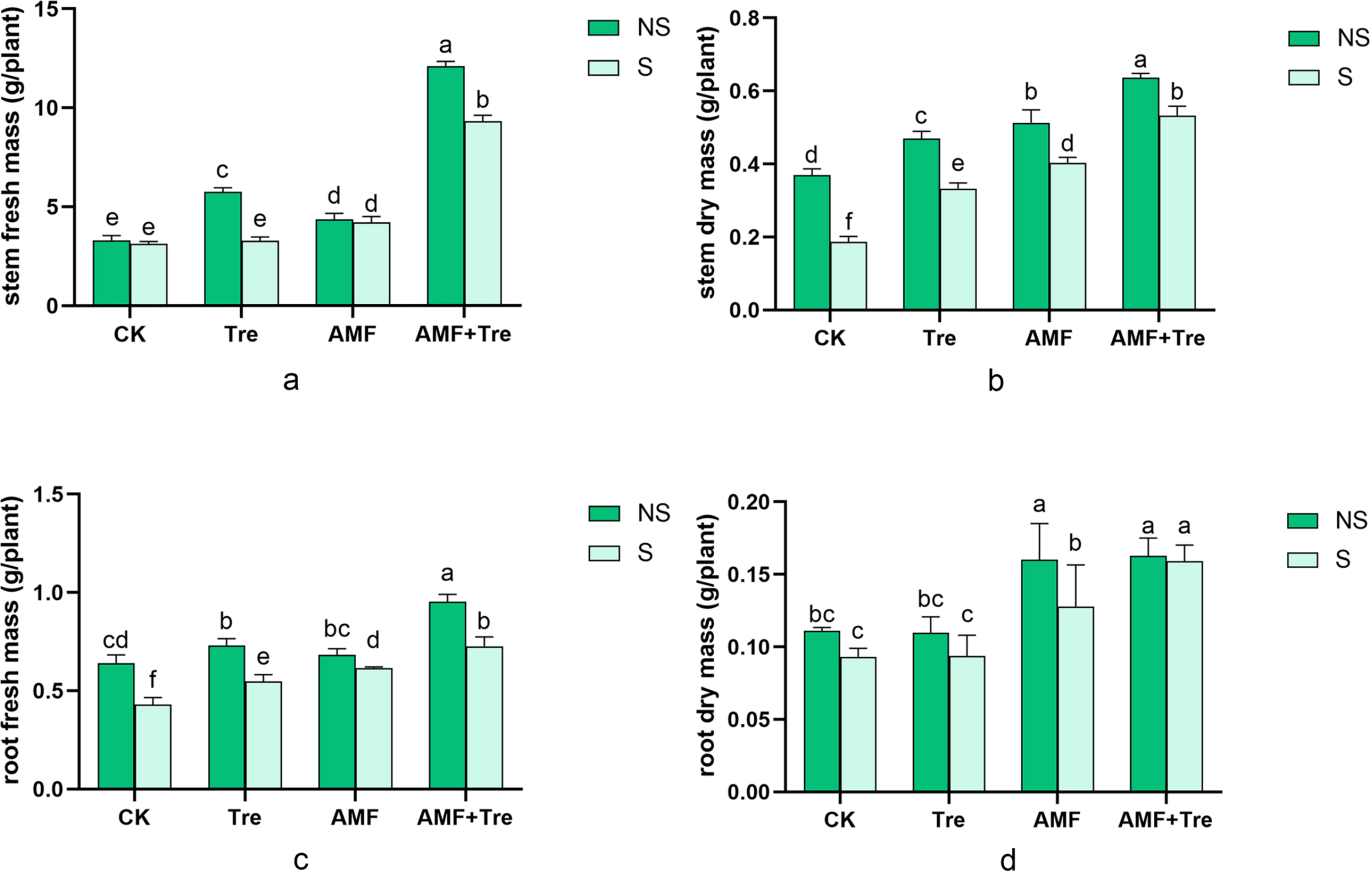
Effects of different treatments on stem (a) and root (c) fresh mass; stem (b) and root dry mass (d) under non-salt stress (NS) and salt stress (S). CK: tomato seedlings without stimulants; Tre: spraying tomato seedlings with trehalose; AMF: inoculated with *C. etunicatum*; AMF + Tre: synergistic treatment of AMF and Tre. The data were expressed as “mean ± standard error” (*n* = 3), and significant differences were measured by Duncan’s test (*P* < 0.05).

Multi-factor ANOVA was performed for fresh mass and dry mass of stem and root in tomato seedlings. The AMF + Tre treatment was the most significant in promoting biomass under salt stress or no salt stress (*P* < 0.05) (Fig. 3). In particular, the stem fresh mass was increased by nearly two times in AMF + Tre treatment in response to salt stress (Fig. 3a).

### Effects of AMF and Tre on root development in tomato seedlings in response to salt stress

**Fig 4:**
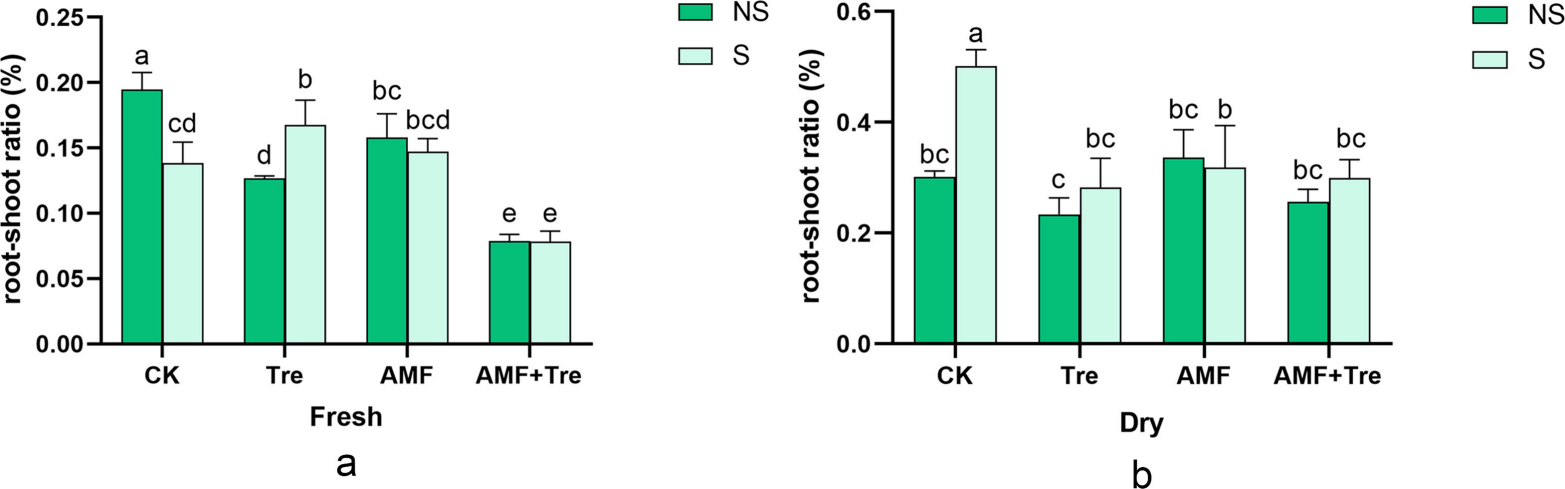
Effects of different treatments on the root-shoot ratio under dry (a) and fresh (b) condition under non-salt stress (NS) and salt stress (S). CK: tomato seedlings without stimulants; Tre: spraying tomato seedlings with trehalose; AMF: inoculated with *C. etunicatum*; AMF + Tre: synergistic treatment of AMF and Tre. The data were expressed as “mean ± standard error” (*n* = 3), and significant differences were measured by Duncan’s test (*P* < 0.05).

Under salt stress, the AMF + Tre treatment significantly reduced the root-shoot ratio (fresh and dry) in tomato seedlings (*P* < 0.05) (Fig. 4a and b). Only Tre or AMF treatment and synergistic AMF + Tre treatment were the better way for increasing above-ground dry weight without increasing root volumes under salt stress compared to no salt stress, which indicated that AMF and Tre play an important role in above-ground nutrient transportation and accumulation (Fig. 4b). On the other hand, synergistic AMF + Tre was the best treatment for reducing fresh root-shoot ratio, which illustrated the synergistic effects of AMF + Tre on strongly accelerating water uptake and transportation from saline soil (Fig. 4a).

**Fig 5:**
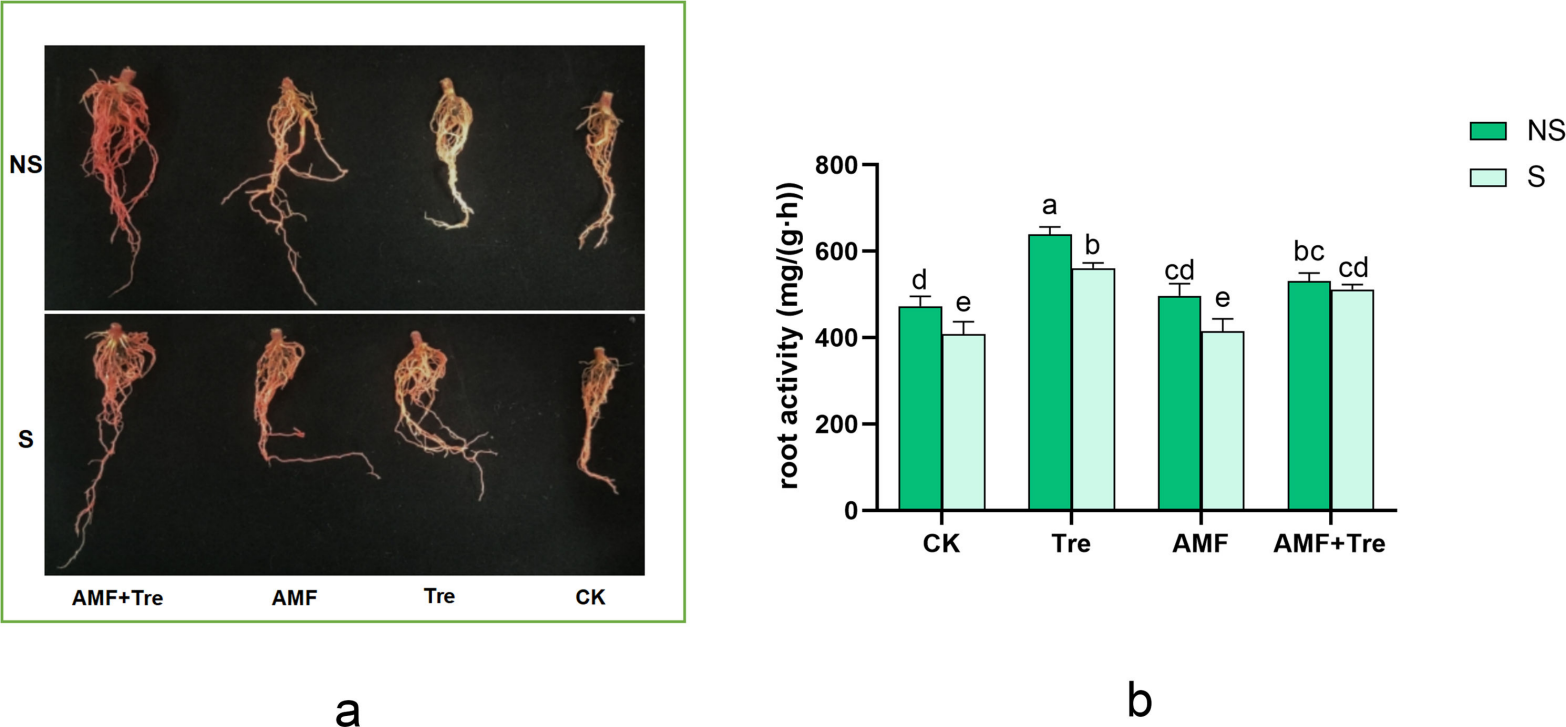
Effects of different treatments on the root activity under non-salt stress (NS) and salt stress (S). (a) qualitative performance of dehydrogenase, showing red root tips or not and (b) quantitative difference of root activity. CK: tomato seedlings without stimulants; Tre: spraying tomato seedlings with trehalose; AMF: inoculated with *C. etunicatum*; AMF + Tre: synergistic treatment of AMF and Tre. The data were expressed as “mean ± standard error” (*n* = 3), and significant differences were measured by Duncan’s test (*P* < 0.05).

Tre produced the most obvious effect on the improvement of root activity under salt stress compared with other treatments, significantly increasing by 37.20% (*P* < 0.05), followed by the AMF + Tre treatment, increasing by 25.28% (Fig. 5b).

### The promoting effect on chlorophyll synthesis in tomato seedlings by AMF and Tre in response to salt

**Fig 6:**
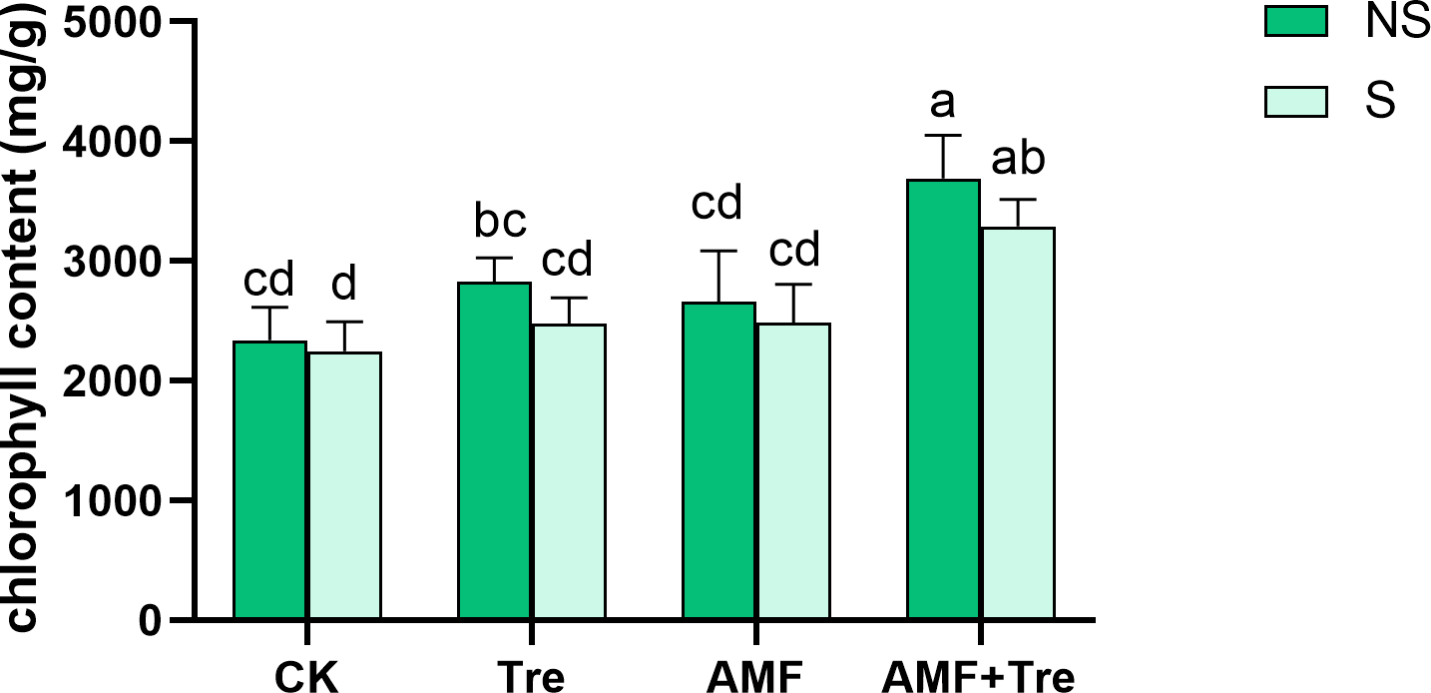
Effects of different treatments on chlorophyll content under non-salt stress (NS) and salt stress (S). CK: tomato seedlings without stimulants; Tre: spraying tomato seedlings with trehalose; AMF: inoculated with *C. etunicatum*; AMF + Tre: synergistic treatment of AMF and Tre. The data were expressed as “mean ± standard error” (*n* = 3), and significant differences were measured by Duncan’s test (*P* < 0.05).

Salt stress caused the reduction of chlorophyll content, while Tre treatment and AMF treatment increased chlorophyll content by 10.18% and 10.49%, respectively, under salt stress, but not significantly (*P* > 0.05). However, the AMF + Tre treatment significantly increased chlorophyll accumulation by 46.24% under salt stress (*P* < 0.01) (Fig. 6).

### Effects of AMF and Tre on antioxidant substance (MDA) in tomato seedlings under salt stress

**Fig 7:**
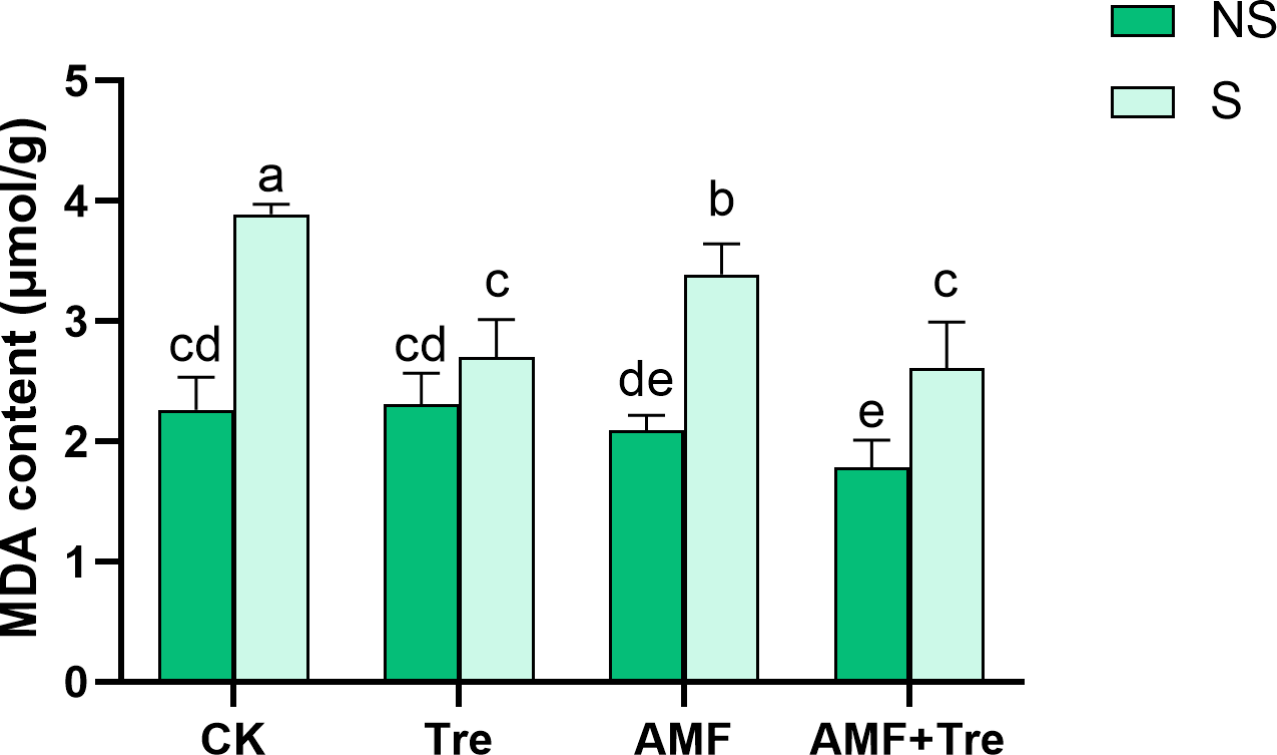
Effects of different treatments on MDA content under non-salt stress (NS) and salt stress (S). CK: tomato seedlings without stimulants; Tre: spraying tomato seedlings with trehalose; AMF: inoculated with *C. etunicatum*; AMF + Tre: synergistic treatment of AMF and Tre. The data were expressed as “mean ± standard error” (*n* = 3), and significant differences were measured by Duncan’s test (*P* < 0.05).

Salt stress caused a significant accumulation of malondialdehyde (MDA), which increased by 71.50% (*P* < 0.05), but Tre treatment, AMF treatment, and AMF + Tre treatment significantly reduced the accumulation of MDA by 32.90%, 12.85%, and 30.59%, respectively (*P* < 0.05) (Fig. 7).

### Response of osmoregulatory substances in tomato seedlings under salt stress

**Fig 8:**
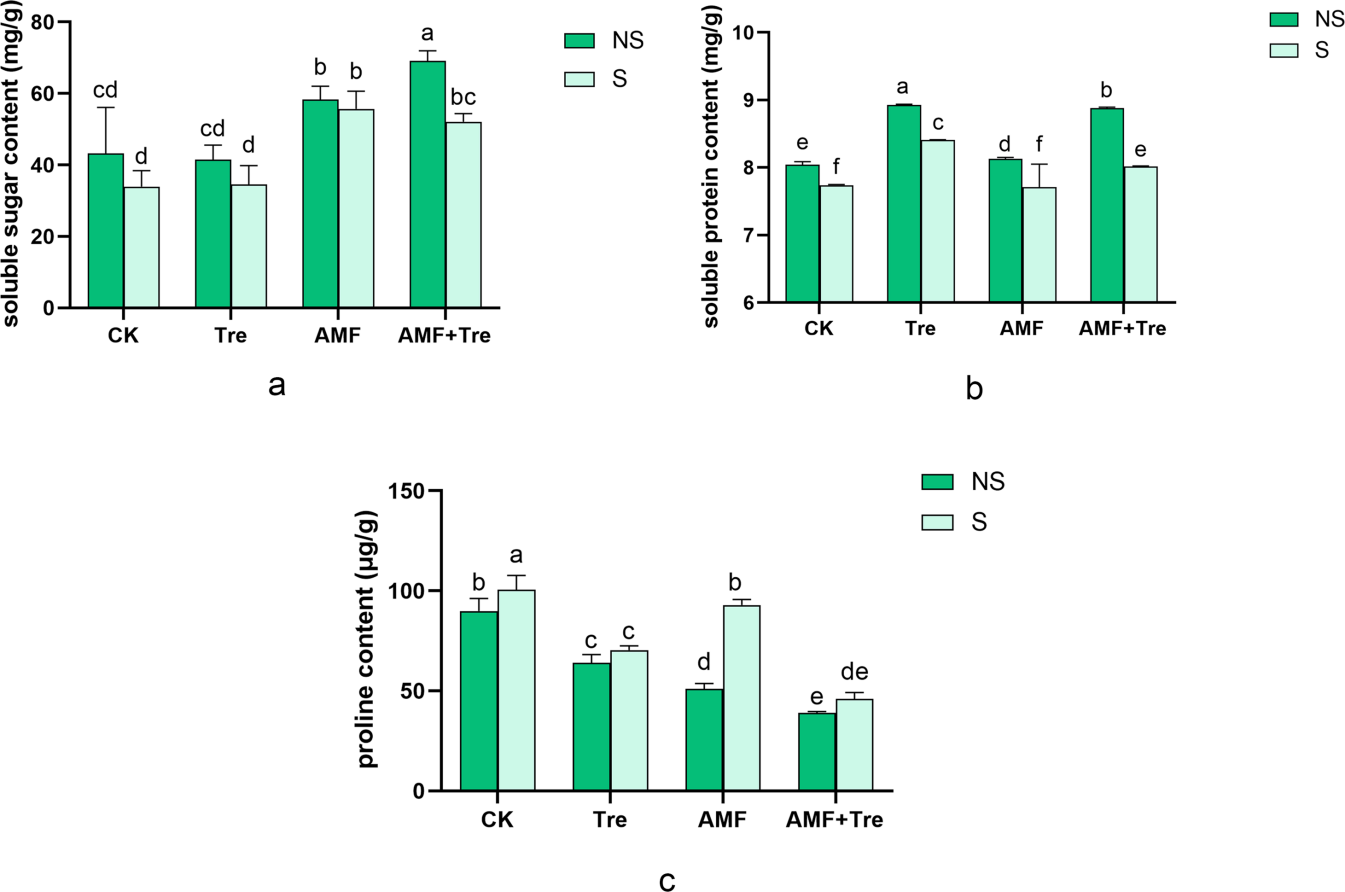
Effects of different treatments on soluble sugar content (a), soluble protein content (b), and Pro content (c) under non-salt stress (NS) and salt stress (S). CK: tomato seedlings without stimulants; Tre: spraying tomato seedlings with trehalose; AMF: inoculated with *C. etunicatum*; AMF + Tre: synergistic treatment of AMF and Tre. The data were expressed as “mean ± standard error” (*n* = 3), and significant differences were measured by Duncan’s test (*P* < 0.05).

Tre treatment, AMF treatment, and AMF + Tre treatment increased the accumulation of soluble sugars under salt stress. The effect of AMF treatment was the best, increasing soluble sugar content by 63.99% (*P* < 0.05). The AMF + Tre treatment increased soluble sugar content by 53.45% (*P* < 0.05). Tre treatment increased soluble sugar content by 1.83%, which did not reach a significant difference level (*P* > 0.05) (Fig. 8a).

The total soluble protein content of all treatment groups ranged from 7.74 mg/g (CK-S) to 8.82 mg/g (Tre-NS). Tre treatment under salt stress produced the most obvious effect of improvement of soluble protein content under salt stress compared with other treatments, increasing by 8.63% (*P* < 0.05), followed by the AMF + Tre treatment (*P* < 0.05) (Fig. 8b).

Salt stress caused the accumulation of proline (Pro), which increased by 11.85% (*P* < 0.05). The AMF + Tre treatment and Tre treatment significantly decreased the Pro content by 54.35% and 29.92%, separately (*P* < 0.05). The effect of AMF + Tre treatment was significantly higher than that of Tre treatment and AMF treatment in decreasing the Pro content (*P* < 0.05) (Fig. 8c).

### Response of antioxidant enzymes in tomato seedlings under salt stress

**Fig 9:**
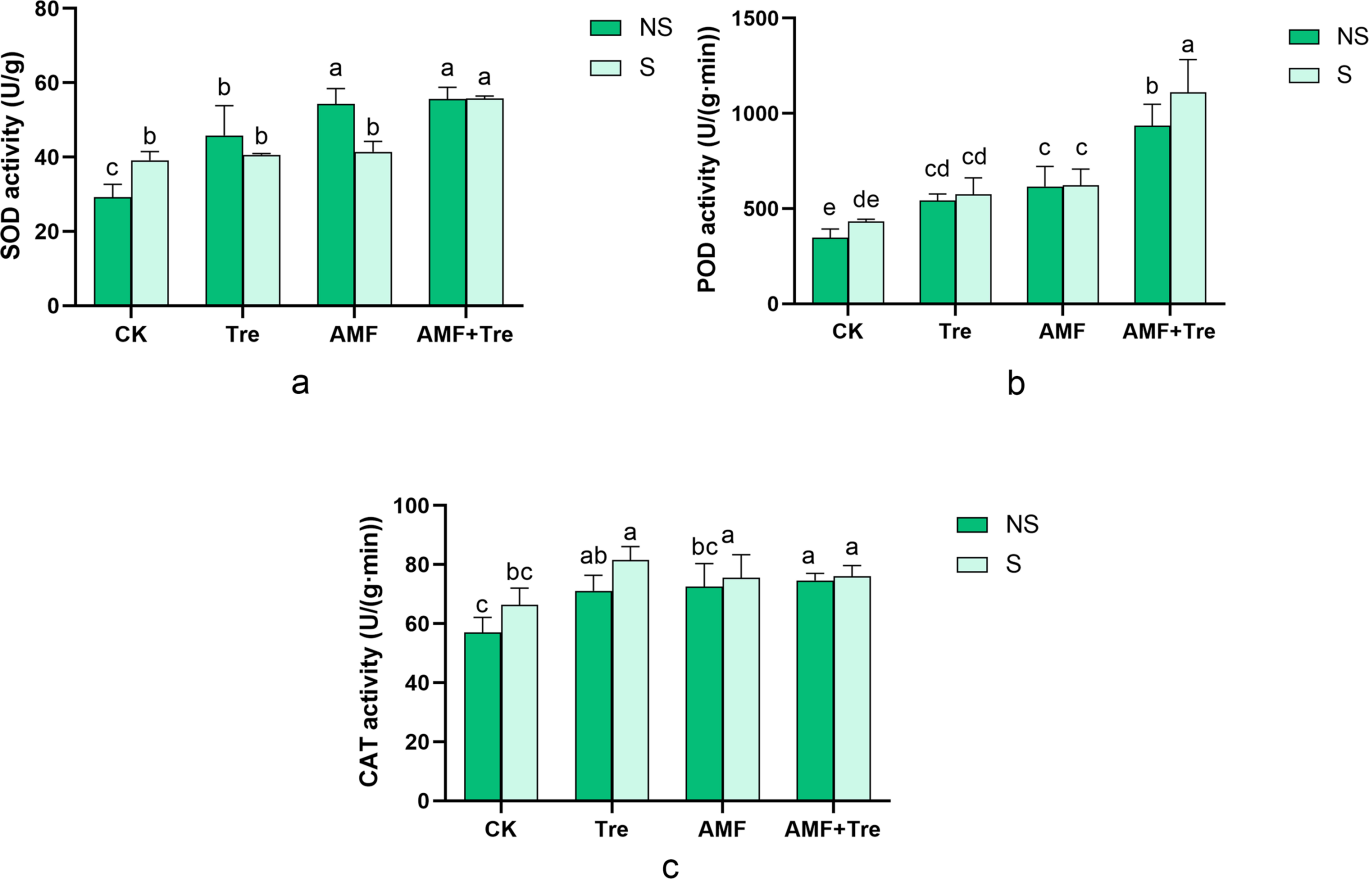
Effects of different treatments on SOD activity (a), CAT activity (b), and POD activity (c) under non-salt stress (NS) and salt stress (S). CK: tomato seedlings without stimulants; Tre: spraying tomato seedlings with trehalose; AMF: inoculated with *C. etunicatum*; AMF + Tre: synergistic treatment of AMF and Tre. The data were expressed as “mean ± standard error” (*n* = 3), and significant differences were measured by Duncan’s test (*P* < 0.05).

Salt stress increased the activity of antioxidant enzymes, including superoxide dismutase (SOD), peroxidase (POD), and catalase (CAT). Under salt stress, the AMF + Tre treatment in seedlings significantly increased the activity of SOD, POD, and CAT, by 42.48%, 157.48%, and 14.50%, respectively (Fig. 9a through c). The activity of SOD under AMF + Tre treatment was 37.36% higher than that under Tre treatment and 34.54% higher than that under AMF treatment. The activity of POD under AMF + Tre treatment was 93.14% higher than that under Tre treatment and 78.71% higher than that under AMF treatment (Fig. 9b).

### Correlation analysis of physiological parameters, AMF colonization, and Tre in tomato seedlings under non-salt stress and salt stress

**Fig 10:**
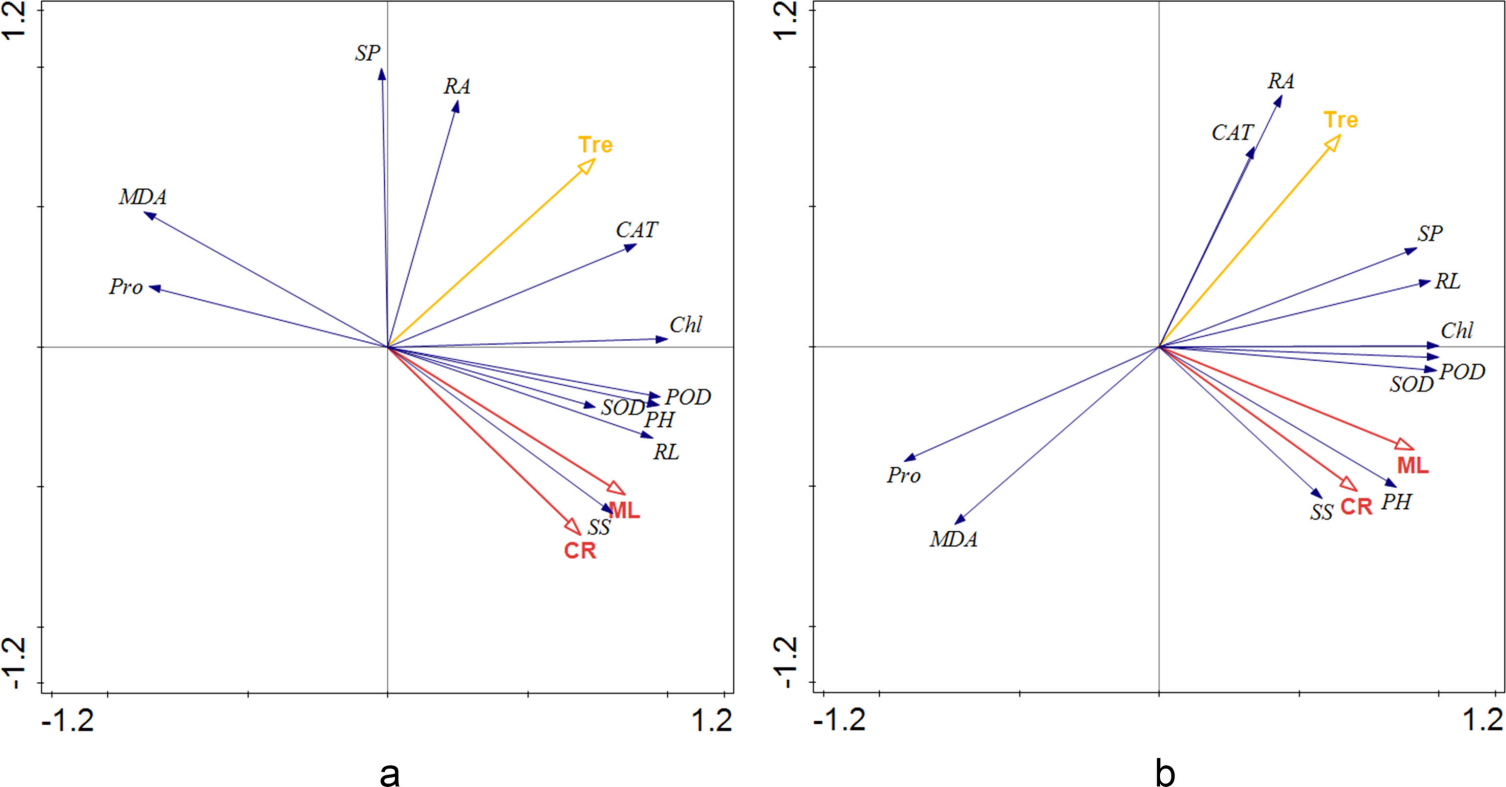
RDA of tomato seedlings under non-salt (a) and salt stress (b). CR: colonization rate, ML: external mycelium length, Tre: trehalose, SP: soluble protein content, SS: soluble sugar content, PH: plant height, RL: root length, Chl: chlorophyll content, RA: root activity, POD: peroxidase activity, SOD: superoxide dismutase activity, CAT: catalase activity, Pro: proline content, MDA: malondialdehyde content.

The RDA of all groups was analyzed by CANOCO 5. The results showed that most indicators were positively correlated with mycelial length, colonization rate, and Tre (Fig. 10a and b). Root activity, CAT activity, and soluble protein content had a more positive correlation with Tre than with AMF colonization. On the other hand, soluble protein content, plant height, and the activity of SOD and POD had a more positive correlation with AMF colonization rate and mycelium length than with Tre stimulant. Salt stress enhanced the correlation between Tre and root activity, while the correlation between soluble sugar content and AMF was also more compact at the same condition. The contents of MDA and Pro were exactly negatively correlated with AMF under non-salt stress, but it was different under salt stress for extremely negative correlation with Tre. The activity of SOD and POD strongly relied on root length and plant height under non-salt stress, while these two oxidases were extremely correlated with chlorophyll stimulated by above-ground photosynthesis under salt stress.

## DISCUSSION

### Synergistic AMF + Tre treatments promoted tomato seedling growth and development in response to salt stress

Salinization not only causes stunting of plant growth but also reduces yield (38–40). Salt stress damages plants in various ways, such as disrupting the ion balance in plants directly and affecting the osmotic balance of plants (41). Unstable microbial community structure and soil aggregates caused by salt (42) further damage plant growth and development.

This study showed that salt stress reduces plant height and root-shoot biomass, which accelerated the penetration sensitivity and root water loss in tomato seedlings. Both AMF and Tre play an important role in improving plant growth and productivity aboveground under salt stress. Nevertheless, besides the benefits mentioned above, the synergistic AMF + Tre treatment not only makes roots retain the moisture but also accelerates the water uptake from saline soil through a well-developed external mycelium net. Under salt stress, Tre treatment had the best promotion effect on soluble protein and CAT activity among all treatments. RDA also showed a strong positive correlation between soluble protein content and CAT activity under salt stress, indicating that Tre reduced salt damage by regulating osmotic substances and antioxidant system.

### Synergistic AMF + Tre treatments strongly improved substance synthesis in tomato seedlings in response to salt stress

Salt stress not only affects the appearance of tomato seedlings but also causes excessive accumulation of free radicals and reactive oxygen species in plants, which damages the integrity of plant cells and reduces plant photosynthesis (43–45). AMF have higher requirements for the accumulation of photosynthetic products in plants, which leads to a large amount of chlorophyll and soluble sugar accumulation in plants. Salt stress reduces the photosynthesis efficiency in tomato seedlings, and the application of synergistic AMF + Tre promotes photosynthesis in tomato seedlings through the accumulation of sugars, which was demonstrated in Giri and Mukerji’s study in 2004 (46).

Plants accumulate some soluble substances to regulate osmotic pressure in response to external environmental conditions when plants are exposed to external stress (47). Our experiments confirmed that salt stress made plants adapt to the stressed environment by synthesizing soluble substances (48, 49). Our study showed that Tre treatment increased soluble protein content efficiently under salt stress, indicating that Tre is the exogenous stimulant that increases stress resistance by improving the efficiency of water use in plants (50). As one of the important substances regulating osmosis, soluble protein also participates in the regulation of plant water. Therefore, exogenous Tre had a greater impact on soluble proteins when regulating the use of water. Therefore, exogenous Tre has a great impact on the use of water by regulating soluble proteins in tomato seedlings.

Salt stress causes lipid film peroxidation to produce MDA (51), and in this process, the plant body also accumulates free Pro to regulate plant function and cope with stress. Under salt stress, MDA content was significantly reduced by Tre treatment and the synergistic effects. Different from the results of Zhou (52), AMF treatment in this study did not significantly reduce the content of MDA. However, the synergistic effects effectively reduced the accumulation of MDA and reduced the toxic effect of salt stress on cells and cell membranes. At the same time, the synergistic effects significantly reduced the degree of stress experienced by plants, thereby reducing the accumulation of Pro. This is also confirmed by RDA, which indicated that applying AMF and Tre could inhibit the synthesis of MDA and Pro under salt stress (Fig. 10b).

### Synergistic AMF + Tre treatments increased antioxidant capacity in tomato seedlings in response to salt stress

When plants are subjected to salt stress, active oxygen metabolism is enhanced. In severe cases, reactive oxygen species cause plant death (53, 54). The activity of antioxidant enzymes is closely related to plant stress resistance and reflects the changes in plant metabolism over a certain period. Antioxidant enzymes protect plants to resist external aggression (55), which mainly include SOD, POD, and CAT. The expression of antioxidant enzymes is commonly induced by salt stress (4, 56). Consistent with the study of Benavides, NaCl increased the activity of POD and CAT (57–59). The results indicated that the AMF + Tre treatment produced synergistic effects at 100 mM NaCl. SOD enzyme catalyzes O_2_^-^ to H_2_O_2_, and then CAT and POD catalyze H_2_O_2_ to H_2_O (60). The intra-group differences of POD activity were not significant, indicating that POD activity was mainly caused by exogenous treatment such as AMF or Tre and was less affected by saline. The activity of SOD, POD, and CAT was positively correlated under salt stress or non-salt stress according to RDA. Compared with Tre treatment, AMF treatment and AMF + Tre treatment had a stronger effect on POD activity, and the RDA also shows that POD activity had a stronger positive correlation with mycelium length than Tre, confirming that POD activity was more susceptible to AMF inoculation. Therefore, AMF induced an increase of antioxidant enzyme system activity in plants with salt stress, and synergistic AMF + Tre treatment significantly increased the physiological adaptability of these plants under salt resistance.

### Conclusion

AMF play an important role in improving plant resistance, and Tre has a non-specific protective effect on biological activity. Our studies indicated that AMF treatment, Tre treatment, and synergistic AMF + Tre treatment promoted the root development and organic matter content in tomato seedlings and further improved the physiological metabolism level in tomato seedlings. Meanwhile, AMF treatment, Tre treatment, or synergistic AMF + Tre treatment reduced the MDA content and improved the antioxidant capacity in tomato seedlings to protect plant cells and alleviate oxidative stress damage in response to salt. The synergistic AMF + Tre treatment was more efficient than separate treatments, and synergistic treatment increased the plant adaptability against salt damage by enhancing cell osmotic protection and cell antioxidant capacity.

## MATERIALS AND METHODS

### Cultures

The AMF species, *C. etunicatum*, was provided by the Institute of Plant Nutrition, Resources and Environment, Beijing Academy of Agriculture and Forestry Sciences. The spore density was 400/100 g soil, measured by the wet sieve precipitation method (61).

The tomato seeds were soaked in 70% ethanol for 30 s and then disinfected with 3% sodium hypochlorite solution for 3 min for surface disinfection. After rinsing with distilled water for three to five times, it was placed on a Petri dish covered with double filter paper and cultured in a constant temperature incubator at 27°C. When more than 90% of the seeds grew to 5 mm small buds, we moved the seeds into plastic pots (49 cm × 20 cm × 14 cm), which were preloaded with 4 kg of autoclaved (0.11 MPa, 121°C, 2 h) substrate (soil:sand:vermiculite:nutrient soil = 2:2:1:1, vol/vol/vol/vol) with 20 g of AMF inoculum.

### Experimental design

**Fig 11:**
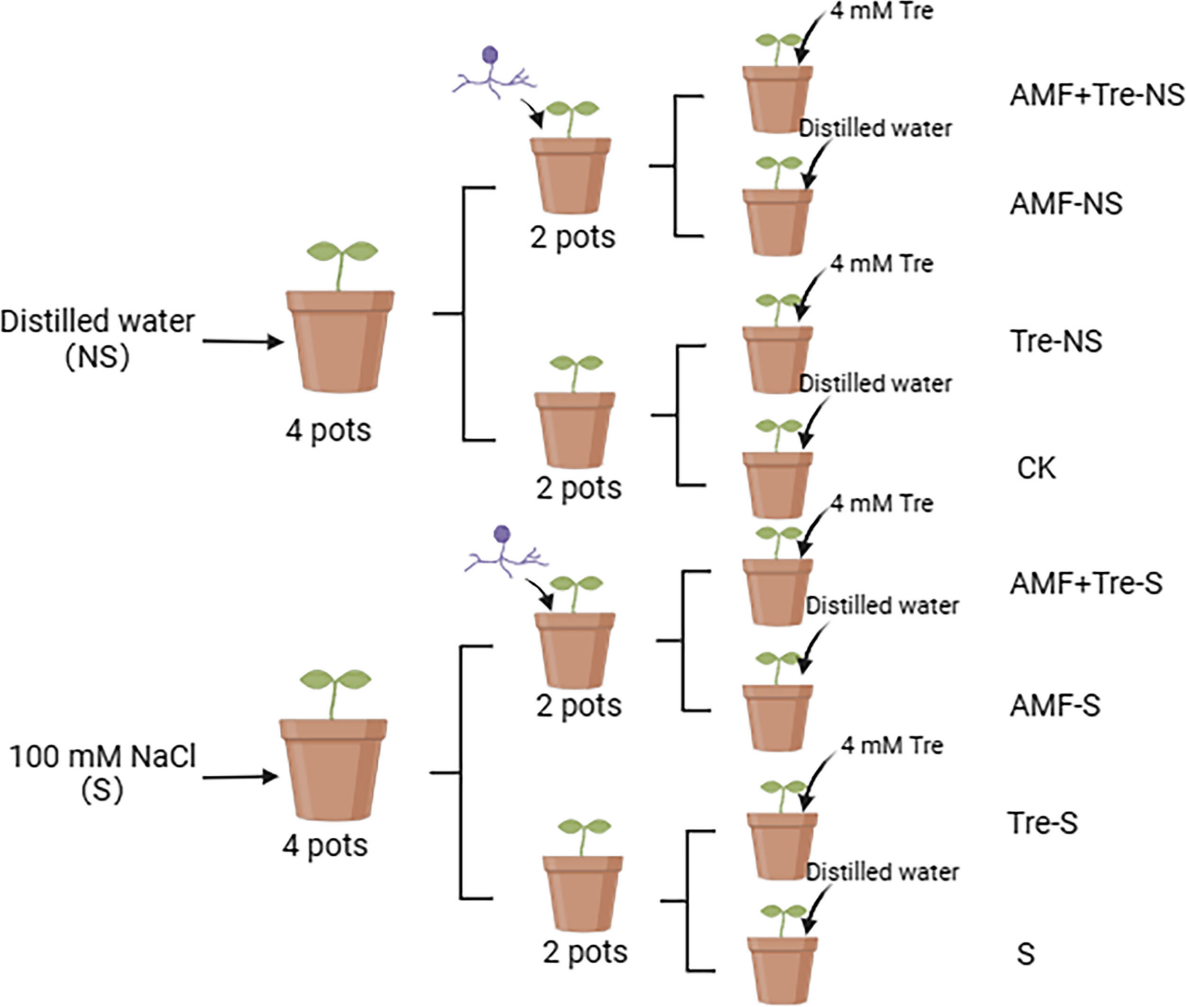
Grouping diagram of this experiment (adapted from BioRender.com)

Figure 11 shows that the experiment was divided into two parts: one poured with distilled water (NS) and the other poured with 100 mM NaCl solution (**S**). The two parts consisted of four treatments (13): tomato seedlings with no additional treatment (CK) (5); tomato seedlings inoculated with *C. etunicatum* (AMF-NS; AMF-S) (62); tomato seedlings sprayed with 4 mM Tre (Tre-NS; Tre-S) (63); and tomato seedlings inoculated with *C. etunicatum* and sprayed with 4 mM Tre (AMF + Tre NS; AMF + Tre S).

**Fig 12:**
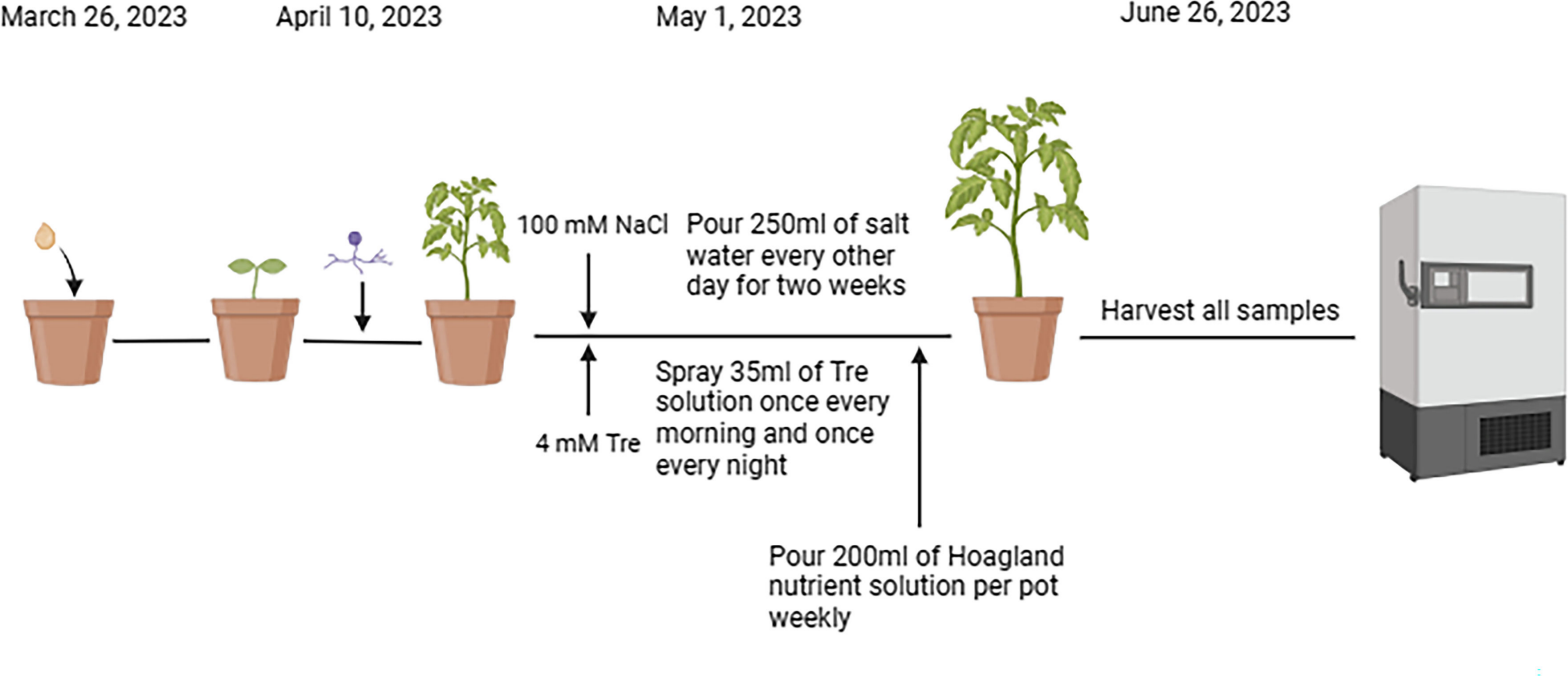
The experimental process diagram of this experiment (adapted from BioRender.com).

The greenhouse trial began on March 26, 2023. When the seedlings formed a true leaf, tomato seedlings were then inoculated with *C. etunicatum*. Salt treatment (100 mM NaCl) was started on 1 May, performed seven times, and poured 250 mL every other day for 2 weeks. The other half was poured with 250 mL distilled water. And, at this time, the 4 mM Tre solution was sprayed into each pot with 35 mL every morning and evening until the end of the test. The non-Tre group was sprayed with 35 mL distilled water. Watering was done every 4 days, and each pot was poured with 200 mL of Hoagland nutrient solution weekly (the formulation of the Hoagland solution is shown in Tables 1 and 2). Each treatment consisted of one pot with 12 replicates, and all samples were harvested on 26 June 2023 (Fig. 12).

**TABLE 1:** Formulation of the individual component concentrates (without ferric salt concentrated solution) that constitute the Hoagland nutrient solution

Name	Content (g/100 mL)	Consumption (mL/L)
Calcium nitrate tetrahydrate	23.6	5
Potassium nitrate	10.1	5
Potassium dihydrogen phosphate	2.72	5
Magnesium sulfate heptahydrate	24.7	5
Boric acid	0.29	1
Manganese (II) sulfate tetrahydrate	0.19	1
Zinc sulfate heptahydrate	0.02	1
Ammonium molybdate tetrahydrate	0.01	1
Copper sulfate pentahydrate	0.01	1

**TABLE 2:** Formulation of the ferric salt concentrated solution^*a*^

Name	Content (g/100 mL)	Consumption (mL/L)	
Ferrous sulfate heptahydrate	0.75	1	
Ethylenediaminetetraacetic acid disodium salt	0.56		

^*a*^ Annotation: The ferric salt concentrated solution was chelated overnight in a magnetic stirrer, and pH was adjusted to 5.5. The volume of each solution was measured according to Tables 1 and 2, and the remaining volume was prepared with distilled water to form 1 L Hoagland nutrient solution. Finally, the pH was adjusted to 6.0.

### Determination of plant development, spore density, and mycorrhizal colonization

After 76 days of inoculation with *C. etunicatum*, the plant height, root length, and fresh weight of the stem and root in tomato seedlings were measured. The roots and shoots were dried for 30 min at 105°C, and then dried the samples until the quality was unchanged at 70°C. Then we weighed it and calculated the root-shoot ratio. After cutting the roots, we used triphenyltetrazole chloride to measure the root activity in tomato seedlings, and the roots were added into conical flasks. The reaction solution was poured, and the reaction was carried out for 5 h until the roots were clearly red. Then, we observed the samples for quantitative determination. The roots were taken out and ground with ethyl acetate and quartz sand, transferred to a test tube, and added with ethyl acetate until the total volume was 10 mL. The absorbance was read at 485 nm.

The symbiotic structure was observed by alkaline dissociation-acid fuchsin staining. The roots were collected and incubated with 10% potassium hydroxide at 95°C for 1 h. The roots were acidified with acid fuchsin for 2 min and then stained with acid fuchsin on an alcohol lamp several times. Finally, we rinsed the color with lactate glycerin, and the symbiotic structure was observed under a microscope (64).

### Determination of organic matter content and substance synthesis

Chlorophyll was extracted from fresh leaves with the acetone method (65). After adding 0.2 g of fresh leaves to the mortar, we added 0.8 mL acetone and 4 mL 80% acetone solution to fully grind and let it stand for 5 min. The solution was filtered into a 20-mL volumetric bottle, and then the volume was set to the scale line. The absorbance was determined with 80% acetone as a blank at 663 nm and 646 nm.

The content of soluble sugar was determined by the phenol method (66). Leaves (0.2 g) were cut and put into a test tube, extracted in boiling water for 1 hour, and then the solution was filtered into a 25-mL volumetric bottle. Then, 0.5 mL of the liquid was absorbed, and 1.5 mL of distilled water was added to determine the absorbance at 485 nm.

Soluble protein content was determined by the Coomassie brilliant blue method. Leaves (0.2 g) were cut into pieces and put into a mortar, ground, and centrifuged quickly. One milliliter of the supernatant was absorbed, and 5 mL of Coomassie brilliant blue solution was added to a colorimeter at 595 nm (67). The content of Pro in tomato leaves was determined by the sulfosalicylic acid method (63). Take 0.2 g fresh leaves into a test tube. Add 5 mL of 3% sulfosalicylic acid and extract in boiling water bath for 10 min. Afterwards, take 2 mL of the supernatant into another test tube, add 2 mL of glacial acetic acid and 3 mL of ninhydrin solution, and extract in boiling water bath for 40 min. Finally, after the extraction with toluene, the absorbance was determined at 485 nm.

### Determination of active substances

MDA content was determined by the thiobarbituric acid (TBA) method (68). Fresh leaves (0.5 g) were cut and put into a mortar. After adding 1 mL of TBA solution, the mixture was ground and centrifuged quickly. Then, 2 mL of the supernatant was taken and added to 2 mL of 0.6% TBA solution. After extracting the mixture in boiling water for 15 min, the absorbance was determined at 532 nm, 600 nm, and 450 nm.

### Determination of antioxidant enzymes

The leaves were taken out at −80°C, and the liquid was sucked with tissues. After putting the leaves in a mortar, we added the phosphate buffer (0.1 mol L^−1^, pH 7.8) into the mortar. The supernatant was obtained by centrifugation at 10,000 × *g* for 4 min, and the supernatant was used to determine the activity of SOD, POD, and CAT. The methods adopted by Fukami et al., Zhu et al., and Amako et al. were used to measure the activity of SOD (69), POD (70), and CAT (62), respectively.

### Statistical analysis of data

The experimental data were summarized, organized, and calculated by Microsoft Excel 2021. The raw data were tested for homogeneity of variance (Levene’s test) before ANOVA. The results were analyzed by multi-factor ANOVA using SPSS19.0 software. The data were expressed as mean ± standard error (*n* = 3) and were used for evaluation. The significance of the difference between the means was determined by Duncan’s multiple range test (*P* < 0.05). The figures were created using GraphPad Prism9.5.
